# Can a Theory of Content Rely on Selected Effect Functions? Response to Christie, Brusse, *et al*.

**DOI:** 10.1080/24740500.2024.2370625

**Published:** 2024-10-06

**Authors:** Nicholas Shea

**Affiliations:** University of London; University of Oxford

**Keywords:** selected effect functions, representational content, evolutionary correctness condition, frequency-dependent selection, evolutionary bet-hedging

## Abstract

In the target article, Christie, Brusse, *et al*. argue that selected effect functions do not, in general, explain why a trait exists in a population and, therefore, theories of representational content should not rely on selected effect functions. This response focuses on the claim about functions-for-representation. The role of evolutionary functions in a theory of content is to pick out outcomes that have been systematically stabilized by natural selection. Correctness conditions are conditions involved in explaining how that happened. Selected effect functions can play that role in the complex equilibria that Christie, Brusse, *et al*. identify. Non-equilibrium cases are also discussed.

## 1. Introduction

In the target article, Christie, Brusse, *et al*. [2022] argue that the explanatory merits of selected effect functions are limited to the very simplest evolutionary scenarios. In more realistic evolutionary scenarios, selected effects do not explain why a trait exists in a population. This, they argue, is a particular obstacle to a theory of content relying on selected effect functions. A central problem is frequency-dependent selection: cases where the way one trait evolves under natural selection depends on the frequency of it and other traits in the population.

Christie, Brusse, *et al*.’s challenge is welcome. They are right to note that selected effect theories of function have largely been explicated by reference to simple evolutionary scenarios. Work is needed to clarify how selected effect functions arise in cases of frequency-dependence, mixed equilibria, and bet-hedging; also to be clear about what explanatory work selected effect functions can perform.

Christie, Brusse, *et al*.’s argument has implications beyond philosophy of biology since their critique has the potential to undermine the way theories of representational content have relied on evolutionary functions. In my own work I have, following Ruth Millikan and David Papineau [Millikan 1984; Papineau 1984], relied on a naturalized notion of function as part of a theory of mental representation. My aim was to home in on a notion of function that is useful for theorizing about representational content—functions-for-representation—rather than trying to say anything about biological functions [Shea 2018]. This follows Millikan’s original motivation for introducing the category of proper functions [Millikan 2022]. So in my response here I will focus on Christie, Brusse, *et al*.’s [2022] argument that theories of content cannot and should not rely on selected effect functions. I largely agree with the points about biological functions made by Samir Okasha [2022], but my claims about function-for-representation do not depend on selected effects function being the right way to understand biological function.

In Shea [2018], I lay out a naturalistic theory of subpersonal representational content in cognitive science. The theory is pluralistic, so it is actually a collection of several accounts of how content is constituted. The accounts are formulated in a common framework and all rely on functions. The pluralistic notion of function I rely on I call *task function*. Task functions capture, in some way, both elements of a broadly Aristotelian approach to teleology, namely: a functional outcome is a natural occurrence that comes about always or for the most part; and a functional outcome is produced for the sake of something or for some purpose. It is this second feature that etiological accounts of function attempt to naturalize. I do so in reliance on the notion of *stabilized function*. Roughly, a stabilized function of a behaviour (or other trait) is an outcome that is produced now because it was produced in the past.

That formulation is too loose to capture the naturally occurring phenomena of interest. I restrict my notion to three more specific cases: natural selection, learning from feedback, and contribution to persistence. The first of these is subject to Christie, Brusse, *et al*.’s critique. An output F from a system S is a stabilized function of this kind just in case ‘producing F has been systematically stabilized by contributing directly to the evolutionary success of systems S producing F’ [Shea 2018: 64]. This is a selected effects account of function, so Christie, Brusse, *et al*.’s challenge is pertinent. Can a theory of content rely on selected effect functions when it is applied in realistic, complex evolutionary scenarios like those involving frequency-dependent selection?

Section 2 sets out the role that functions play in a theory of content. Section 3 argues that selected effects functions can play that role in the complex equilibria that Christie, Brusse, *et al*. identify. Section 4 considers non-equilibrium situations.

## 2. Functions in a Theory of Content

The original reason that theorists of content turned to natural selection was to account for the normativity that seems to attach to representational content. A bee dance which represents that *there is nectar 250 metres away in the direction of the sun* is correct if there really is nectar at that location and incorrect otherwise. Correctness / incorrectness seems to be a normative distinction, so a naturalistic theory of content needs to show how such a distinction can arise in the natural world of descriptive facts. To put it another way, a theory of representational content has to account for the possibility of misrepresentation. The idea was to explicate incorrectness or misrepresentation in terms of malfunction. Natural selection comes into the picture as a naturalistic explanation of the function / malfunction distinction.

My own view is that the correctness / incorrectness that attaches to representational content is not genuinely normative, in the sense of prescribing what one ought to do. That is true at least in cases of subpersonal representational content and in the cases of biological signalling with which Christie, Brusse, *et al*. [2022] are concerned.^1^ Even if correctness / incorrectness is a descriptive distinction, the theorist of content has to show how it arises naturally. Here, the explanatory connection between correct representation and behavioural success is key. Correct representation can explain successful behaviour; misrepresentation, failure. Natural teleology comes into the picture as an account of what counts as successful behaviour.

A distinction emphasized by Millikan and Papineau, and somewhat elided by Christie, Brusse, *et al*. [2022], is also important—the distinction between evolutionary functions and evolutionary success conditions. A function is an effect produced by an organism, or by one of its traits or behaviours. Evolutionary success conditions are conditions that figure in an explanation of how an effect contributed systematically to survival and reproduction. The red-headed finches discussed by Christie, Brusse, *et al*. have a behavioural trait of competing aggressively for nesting cavities. This trait has the evolutionary function of acquiring good nesting sites. It also has an evolutionary success condition: that the resident bird is a dove-ish type that will give up the cavity without exacting a high competition cost. The latter is a condition that obtained on occasions where ancestral red-heads’ aggressive behaviour contributed systematically to their survival and reproduction. When naturalizing descriptive content, it is evolutionary success conditions that take centre stage.

This innovation was a crucial insight [Millikan 1984, 2022]. But we can go beyond the argument that evolutionary success conditions are a naturalistic way of reconstructing the success / failure distinction. There is a deeper reason for the connection to natural teleology [Shea 2018: 48–52]. We need to step back and ask why the notion of representational content is so explanatorily useful. Why is the world arranged in such a way that it affords the characteristically representational form of explanation—explaining success and failure of behaviour in terms of the obtaining or otherwise of some worldly condition? There is a wider pattern here and natural selection is a key part of why that pattern exists. Natural selection is a stabilizing process. It is one way in which traits are spread or preserved over time. (Note: this does not require that it always has this effect.) Outcomes that are the target of selection often come to be produced more robustly: across a wider range of initial and perturbing conditions. Learning is one way of producing outcomes more robustly. It is also a stabilizing process in its own right.

Another commonly adopted tactic for producing important outcomes more robustly is by representing the world and acting appropriately on those representations. To put the point without using the notion of representation, the robustness trick that many organisms have hit upon is to make use of exploitable relations between internal states and features of the environment, in such a way that outcomes which have been the target of stabilizing processes like natural selection and feedback-based learning come to be more robustly produced. There is a natural cluster of properties in the world linking stabilization, robustness, and reliance on these kinds of internal states (i.e., representations). That cluster arose, ultimately, because of evolution by natural selection. It is because these properties cluster together in nature that the notion of representation is so explanatorily useful. This argument does indeed trade on Darwin’s basic insight that one of the reasons for the appearance of teleology in nature is evolution by natural selection [Christie, Brusse, *et al*. 2022: end of §1], but it goes further and identifies a reason why natural selection gives rise to so many systems that are susceptible to representational explanation.

The role for functions in a theory of content is, then, to pick out outcomes that have been the target of stabilization and are robustly produced [Shea 2018: 65]. My notion of task function is designed to combine these two features. Stabilization in virtue of natural selection is one case [Shea 2018: 64, point (i)]. Thus, the role of selected effects functions in a theory of content is to pick out outcomes F that have been systematically stabilized by natural selection. Correctness conditions are conditions involved in explaining how that happened. They arise (roughly) where carrying information about a condition C [Shea 2018: 84], or standing in a structural correspondence involving condition C [Shea 2018: 124], explains how producing outcome F was stabilized by natural selection.

## 3. Complex Equilibria

Does Christie, Brusse, *et al*.’s [2022] critique undermine the strategy of relying on selected effect functions in this way in a theory of content? Christie, Brusse, *et al*. point out that only in the simplest scenarios is natural selection guaranteed to produce adaptation. But selected effect functions do not depend on assuming that natural selection is guaranteed to produce adaptation. They arise only in cases where natural selection has in fact produced adaptation (or something like adaptation). Selected effect functions just require that an outcome has persisted or spread over time because of its positive contribution to the survival and reproduction of the organisms which produce it.^2^ Is it problematic for a theory of content to rely on functions in that sense?

A central plank of Christie, Brusse, *et al*.’s argument is that selected effect functions rarely do a good job of explaining why a trait evolved. They fairly point to a strand in the literature which argued that functions are apt to explain the existence of a trait. This point needs to be handled carefully, however. A first observation is that this would be to put the point the wrong way round. It is not that having an evolutionary function explains the existence of a trait, but rather that where producing an outcome explains the current existence of a trait, the trait thereby has a function.^3^ The idea of explaining existence is also nuanced. We are not concerned with the ultimate origin of the trait, which may lie in random mutation (but see Godfrey-Smith [2012]). It is a matter of explaining why a trait is found in a population (and not necessarily at fixation) (cp. Christie, Brusse, *et al*. [2022: end §2]). So the substance of Christie, Brusse, *et al*.’s objection is that the following are a poor explanandum-explanans pair:

Explanandum. Trait T is found in a population at some (non-zero) frequency f.

Putative explanans. Trait T causes effect or outcome O (where O is one of the effects picked out as a selected effect function of T).

Christie, Brusse, *et al*.’s major complaint is that selected effect functions leave out much of the information that is needed to explain why a trait evolves by natural selection in a certain way, for example why a hawk-type trait evolves to reach a certain equilibrium frequency with respect to a competing dove-type trait. Our question, then, is whether that is a valid objection to the way theories of content rely on selected effect functions. Does the role they play in a theory of content call for this kind of comprehensive explanation of the existence of a trait (at a certain frequency, say)?

I would argue not. My account of content, for example, relies on selected effect functions in order to home in on outcomes that count as successes; and, by extension, the conditions under which producing those outcomes led systematically to survival and reproduction. That is deliberately to rely on just part of the full evolutionary explanation of why a trait evolved. If we were concerned with explaining why a trait evolved and stabilized at a certain frequency, then it would indeed seem arbitrary to focus on its positive contribution to fitness and ignore the circumstances in which it reduced fitness. But the point of selected effect functions in a theory of content is not to encapsulate or recapitulate the full evolutionary story about a trait. It is to capture an aspect of the evolutionary history that is relevant to content. This gives us a principled reason to be concerned specifically with past successes. A theory of content needs just a particular aspect of the evolutionary history—effects that positively contributed to survival and reproduction, together with the conditions whose obtaining explains how they so-contributed. Selected effect functions, and their associated evolutionary success conditions, serve to pick out those properties.

To demonstrate that it is only this particular aspect of the evolutionary history that is needed for a theory of content, I propose go through Christie, Brusse, *et al*.’s problem cases to show that, when the evolutionary scenario is complex, with traits having had both positive and negative effects, for example, involving frequency-dependence, nevertheless selected effect functions home in on the properties that a theory of content needs to have recourse to. Christie, Brusse, *et al*.’s cases do not concern representation, but they argue that the evolutionary scenarios they present will be common in situations where representation evolves. That is certainly true for between-organism signalling. It is less clear that the problems of frequency-dependence and mixed-strategy equilibria will characterize the way within-organism signalling evolves. Most of the cases from cognitive science that I examine in [Shea 2018] are internal to an organism. Where these systems have evolved by natural selection (they also involve learning) that may have been through more straightforward evolutionary scenarios. Nevertheless, the way functions arise and play a role in my theory of content does, I think, apply without modification to more complex evolutionary equilibria.

The signalling cases Christie, Brusse, *et al*. [2022] discuss in section 5 have been extensively studied in evolutionary game theoretic models. Rosa Cao, Peter Godfrey-Smith, and I developed a vector-based notion of functional content for these models, designed specifically to deal with mixed equilibria [Shea, Godfrey-Smith, and Cao 2018]. A functional content vector is a more nuanced way to capture contents than we get by simply giving a correctness condition. It aims to capture, in some way, the relative importance of different world states. For example, if the content of a representation R is <⅔,⅓,0>, R is largely correct if condition C1 obtains, somewhat correct if condition C2 obtains, and wholly incorrect if condition C3 obtains. In what follows I will discuss both the reliance we placed on functions in fixing vector-based content for model signalling systems in Shea, Godfrey-Smith, and Cao [2018], and the reliance I place on evolutionary functions in my (non-vector-based) account of representational content in cognitive science [Shea 2018].

First off: the problem of frequency-dependent selection and mixed equilibria. Christie, Brusse, *et al.*’s [2022] example is the aggressive red-headed finch, whose frequency at equilibrium is explained by the idealized hawk–dove model. To get representation into the picture, suppose the finches have internal vehicles^4^ R1 and R2 that correlate with conspecifics and nest sites, respectively. They also have an internal vehicle R3 that prompts attacks on the organism detected. Suppose too that red-headed finches have the disposition to token^5^ R3 when R1 is tokened together with R2. As a result, they attack conspecifics at nest sites. Black-headed finches, by contrast, are not disposed to token R3 when R1 and R2 are tokened together. Natural selection stabilizes the population at a mixed equilibrium with a certain proportion of the aggressive red-heads and the passive black-heads.

The interaction of R1, R2, and R3 produces an outcome O in red-headed finches, namely aggressive behaviour towards conspecifics at nest sites. (The three vehicles are likely to be involved in other kinds of behaviour as well. That will be important to their having determinate contents, but does not raise issues that need detain us here.) Producing O has led systematically to survival and reproduction in ancestor red-headed finches. There are finches around producing O today in part because ancestors produced O in the past. None of that requires that the trait has gone to fixation, nor that it always contributes to survival and reproduction. The occasions where it has systematically so-contributed are privileged, however. They underpin stabilized functions (hence task functions).

Now to contents. We look at the way correlational information carried by R1, R2, and R3 enters directly into the explanation of how outcome O was systematically stabilized. Producing O only led systematically to survival and reproduction when two conditions obtained: there was a good nest site, and the bird guarding it was a passive conspecific. Thus, in the idealized situation I have described, it is because R1 correlates with the presence of passive conspecifics (black-heads), and R2 with good nest sites, that producing O led systematically to survival and reproduction. Those are correlations at input. On the output side, the fact that tokening R3 correlates with producing aggressive behaviour (outcome O) also figures in the explanation. So R1 has the descriptive content *passive conspecific present*, R2 has the descriptive content *good nest site present*, and R3 has the directive content *act aggressively*.^6^

We are assuming that the mix of hawk-types (red-heads) and dove-types (black-heads) observed in nature is explained by the evolutionarily stable equilibrium in the hawk–dove model. When random variation or other perturbations increased the frequency of black-heads, red-heads increased in fitness and thus in frequency. The converse happened when the frequency of red-heads rose above the equilibrium. At equilibrium, the aggressive behaviour of the red-heads is partly helping them, when they encounter passive black-heads, and partly hindering them, when the encounter other red-heads. The episodes that positively contribute to their survival and reproduction, both in equilibrium and out of equilibrium, are occasions when they encounter a black-head, behave aggressively, and acquire a good nest site. This is true whether the population originally approached the equilibrium from ‘above’, with a population of hawk-types invaded by dove-types, or from ‘below’. Neither the fact that the equilibrium involves a mix of types, nor the fact that fitness is frequency-dependent, make my account of stabilizing function inapplicable. Nor do these complications undermine the way my theory relies on functions in fixing content.

I turn now to the vector-based functional content defined by Shea, Godfrey-Smith, and Cao [2018]. That account has no problem handling such cases. Functional contents were designed specifically to apply to the mixed equilibria that are so common in evolutionary game theoretic signalling models. If the internal signals I have just described were modelled this way, the functional content vectors would be <1,0> and <0,1>. That corresponds exactly to the descriptive contents set out above. However, in the paper we show how more complex functional content vectors arise in more complex mixed equilibria. The entries in the content vector capture, for each signal, the relative contribution to the way the sender-receiver behaviours are stabilized made by sending that signal in each world state at that equilibrium.^7^ The paper contains several examples of functional content vectors at mixed equilibria.

The functional content vector also applies straightforwardly to cases of bet-hedging. The sender can have a strategy, in a particular world state, of randomizing between two or more signals in a certain proportion; the receiver can have a strategy, in response to a particular signal, or randomizing between two or more behaviours in a certain proportion. Functional contents apply to the signals, not to the sender’s overall behavioural pattern. So Christie, Brusse, *et al.* [2022] are right to observe that they are not giving us the whole evolutionary story. They are not telling us why senders randomize, and do so in a certain proportion. That is explained by the equilibrium in the evolutionary dynamics. That equilibrium in turn serves to fix functional contents. But functional contents are not intended to recapitulate all relevant aspects of the evolutionary model. They are intended as a compact summary of the way signals are involved with world states so as to stabilize patterns of sending signals and acting on them.

Next consider bet-hedging in relation to my account in Shea [2018]. It will be somewhat artificial to consider representation in the context of bet-hedging, since the whole point is that no information is available about which environment will be faced by the next generation. All offspring will encounter the same environment, but it might be suited to one behaviour or it might be suited to another. Evolution might select a single best cover-all strategy, or it can select for a diversified strategy, a disposition to randomize between different options.

To shoe-horn representation into the picture, consider the seed dormancy example, and let us suppose that there is an internal chemical produced by the arrival of spring. (Maybe it is generated by rewarming after a period of cold.) Seeds can be programmed to react to a single pulse of this chemical—call this signal S1—by germinating. Or they can be programmed to react to two pulses of the chemical (produced by two consecutive springs)—call this signal S2—by germinating, and to do nothing in response to S1. If drought were to occur reliably every second year, we would expect all seeds to be programmed to germinate in response to S2. But what if good years and drought years occur at random? Assuming that seeds are much more likely to be successful in good years than in drought years, then a diversified bet-hedging strategy may evolve.

The diversified bet-hedging strategy consists of a trait T that causes half the seeds to be S1-germinators and half to be S2-germinators (or some other proportions). The trait of being an S1-germinator has a selected effect function. Its function is for the seed to germinate in the first year. An evolutionary success condition is that there is rain in the first year. The trait of being an S2-germinator has a selected effect function: for the seed to germinate in the second year. Its evolutionary success condition is that there is rain in the second year. Signals S1 and S2 have corresponding contents, contents that differ as between the two types of germinator. The functions and associated success conditions pick out the circumstances in which each trait contributed positively to survival and reproduction. As before, this is just a subset of the whole evolutionary story, a story which is richer and can explain the proportions of each type (and will be based on geometric not arithmetic mean reproductive fitness).

We can, however, also ask about the representational content associated with trait T (the disposition to randomize). This is to go beyond the kinds of cases that Christie, Brusse *et al*. [2022] were concerned with, of signalling within the organism, or signalling between organisms in the same generation. Here instead we are focusing on the way DNA carries information down the generations, making available to the developing organism information that has been generated by selection over many generations [Shea 2007b, 2011]. In real biological cases this information may be weighed against and integrated with information that is available to the individual developing organism as the basis for adaptive phenotypic plasticity [Shea, Pen, and Uller 2011; English, Pen, *et al.* 2015]. But in our simple bet-hedging case we are thinking of a genetic variant G that alone causes the parent to have trait T. (Recall that T in turn causes half the seeds it produces to be S1-germiantors and half to be S2-germinators.)

Genotype G carries the information that past environments for germination were sometimes all-drought years and sometimes all-good years. (The bet-hedging strategy would not have evolved, we are supposing, had the seeds been dispersed over a mix of good and drought patches in each generation, because then the geometric mean fitness would not exceed the arithmetic mean.) The parent’s response to G is to produce two types of seeds, half S1-germinators and half S2-germinators. We have already discussed the functions of the specific seed traits (being an S1-geriminator; being an S2-germinator). Now the question is: what is the function of trait T (the parent’s disposition to randomize)? Its selected effect function is to produce a mix of seed types. That is the thing that T has done in evolutionary history that has positively contributed to survival and reproduction. And a condition that was in place and explains why this outcome positively contributed to survival and reproduction is that there were, over the generations, sometimes all-drought years and sometimes all-good years. G carries the information that this is how environments were in the past. So G ends up representing this condition.

In summary, for each trait, selected effect functions are well-suited to the content-determination story. If we are focusing on particular traits—germinating in response to S1, say—then, to give content, we need to home in on just that part of the evolutionary story in which this behaviour contributed positively to survival and reproduction. When we switch to asking about the function of the randomizing trait itself, selected effect functions bring more of the evolutionary picture into the frame. Thus, I would argue, the theory does a good job of ascribing functions and contents to representations to which there is a bet-hedging response.

## 4. Non-equilibrium Cases

Christie, Brusse, *et al*.’s [2022] problem cases concern evolutionary equilibria. In evolutionary models of biological signalling, the notion of functional content has its home in the equilibrium cases [Shea, Godfrey-Smith, and Cao 2018]. The underlying idea is that functional contents capture the way senders and receivers use signals to coordinate behaviour with world states and payoffs. Equilibria are also at the heart of my theory of content in cognitive science [Shea 2018]. My argument about the natural cluster that supports the usefulness of representational explanation (section 2) concerned stabilized outcomes. Evolutionary equilibria are paradigm cases (both signalling equilibria and partial pooling equilibria). As we move to non-equilibrium cases we are moving away from paradigmatically representational explanation. Nevertheless, non-equilibrium cases do have some representation-like features—for example, they can involve the transmission of Shannon information with fitness consequences. So it may be useful to briefly consider the applicability of concepts of evolutionary function to such cases.

It is straightforward to define a functional content vector that applies throughout the space of strategies in a signalling game, both in and out of equilibrium. (This is different from the functional content vector of Shea, Godfrey-Smith, and Cao [2018], which is only defined at equilibrium.) Each signal that can be sent has a functional content. Where senders and receivers have non-coincident interests, there is a separate functional content vector for each. The functional content vector for a signal records, for each world state, the average payoff received in that world state (given sender’s strategy for sending signals in response to world states, receiver’s strategy for acting on signals, and the probability of world states). Where payoffs for sender and receiver differ, their respective functional content vectors will be different. So-defined, functional content is no longer capturing how senders and receivers coordinate to achieve payoffs. It is recording fitness-relevant facts and showing, for each signal, which world states matter most to each party. This captures an important part of what drives the evolutionary dynamics. Unlike correlational information (Shannon information), it captures something of the functional significance of the signals. But it is some distance from the core idea of representational content which, as I argued above, has its home in stabilized equilibria and coordinated behaviour.

Jonathan Birch has argued that content should be ascribed to out-of-equilibrium situations by reference to the equilibrium towards which the evolutionary dynamics is heading [Birch 2014]. The content of a signal is the information it would carry at the nearest separating equilibrium in the dynamics. That seems to me problematic for the same reason that forward-looking accounts of function are problematic [Godfrey-Smith 1994; Artiga 2014; *pace* Griffiths 2009]. The idea of evolution following a path through a fixed landscape is an idealization of the model. In practice, the evolutionary trajectory followed by a population depends on all kinds of contingent factors. Contingent factors can also affect the shape of the evolutionary landscape itself, which thus changes over time in a way that is contingent on what actually happens at each time, not just on the shape of the foregoing landscape. If we ask about the actual history of an organism or a population, there is a determinate way that things actually unfolded. A future-directed notion of the equilibrium a population is heading towards is much more open-ended. Birch’s notion may be applicable in some circumstances, where endogenous and exogenous factors conspire to determine a clear future evolutionary trajectory, but it is likely to be a misleading idealization in many realistic out-of-equilibrium evolutionary situations.

A related, less problematic, idea is to turn to local evolutionary gradients. What is evolution doing at a given point in the dynamics to change the frequency of traits in the population? That would allow us to define functional content vectors. These would not simply record average payoffs, but contribution to change. There is a local rate of change for each type (each type of sender, each type of receiver). Signals may well be involved in producing that change. Each entry in the functional content vector would record the average contribution of sending that signal in each world state to the change in frequency of that type. That avoids the objection about the open-endedness of the future evolutionary trajectory. Functional content vectors, so-defined, would capture something important about the functional significance of signals-in-world-states. Again, however, this bears only a family resemblance to the core notion of representational content. Nor would it give a complete story about the evolutionary dynamics, as Christie, Brusse, *et al*. [2022] seem to want, although it could prove to be a useful compact summary of how signals are enmeshed in the local dynamics.

Finally, I want to consider out-of-equilibrium cases where the population follows a cycle that keeps within a circumscribed portion of the dynamical landscape (cp. Okasha [2022]). Brian Skyrms describes the case of the rock–paper–scissors game, which models many natural cases, and also arises frequently in signalling games [Skyrms 2010: 57–60, 77–9].^8^ The frequencies of three different types continually change as A does better than B but loses out to C, and so on. Functions will come into the picture to explain how outcomes contribute to keeping the frequency of a type within a certain region of state space. Why are there individuals around today with trait A? Answer: because of effects that trait A has had in the past, as the population moves around the cycle. In the case of A, that is because of the way it has out-competed B, despite also losing to C. These competing forces sometimes lead to an increase in the frequency of A, sometimes to a decrease, depending on the relative frequencies. But the effect that A has that keeps it within this region, rather than going out of existence, may be consistent throughout the cycle. If so, that would be a basis for ascribing stabilized functions. If, further, these outcomes were robustly produced, then we would have task functions, and hence a basis for the application of the framework in Shea [2018]. So there is some prospect of a selected effects notion of function being useful for theories of content in out-of-equilibrium cases when they involve cycles.

In short, the idea that connects content to stabilization does apply to evolutionary cycles that remain within a circumscribed portion of phenotypic space, as opposed to trajectories that are chaotic or move through the adaptive landscape in a contingent, open-ended fashion. Furthermore, there are two content-like properties that are available throughout the selective landscape: a generalized functional content vector and a vector of local evolutionary gradients.

## 5. Conclusion

Christie, Brusse, *et al*. [2022] have provided a valuable critique of selected effect functions. Their challenge forces the theorist of content to be clearer about what explanatory work selected effect functions can and cannot do. Such functions are deliberately selective. They appeal to just one aspect of what is often a complex evolutionary story. This restricted focus—homing in on positive contribution to fitness and the circumstances in which that was attained—is justified by the role that functions play in a theory of content. It is just what a theory of content needs.

## Footnotes

1 This qualification applies throughout. When it comes person-level beliefs and desires, linguistic contents, and the reasons we offer to one another as justifications, a different kind of normativity may be in play.

2 Note that a trait T’s having had higher relative fitness does not guarantee that this condition is met. A variant T with higher relative fitness may produce an outcome O that decreases the fitness of its bearers, as well as of other variants, driving the population towards extinction. Producing outcome O would not, then, qualify as a stabilized function. It would not figure in an explanation of why trait T is present in the population today. (Thanks to Peter Takacs for the example.)

3 It may be that the so-constituted evolutionary function can also explain the current production of the outcome—*pace* worries about a Euthyphro problem [Godfrey-Smith 1996; Shea 2007a]—but that is not the way selected effect functions are constituted.

4 I use ‘vehicle’ when picking out a representation non-semantically. A vehicle is an internal state or process that carries content.

5 A vehicle R is *tokened* when internal state or process R occurs or is realized.

6 The contents of R1 and R2 may be different if they are also involved internal transitions conditioning other forms of behaviour. Note also that capturing contents with natural language sentences misleadingly suggests a level of determinacy, and a kind of constituent structure, that the unstructured representations do not have.

7 Griffiths and Matthewson [2018] do something similar when they argue that we need to record the positive and negative contributions of a trait to fitness separately at different stages in the life history of an organism.

8 Jonathan Birch discussed cases of this kind in ‘Functions and Cycles’, an oral presentation at the British Society for the Philosophy of Science Annual Conference, July 2022.
